# Mid-term outcomes of a smoking cessation program in hospitalized patients in Türkiye

**DOI:** 10.18332/tid/191239

**Published:** 2024-07-30

**Authors:** Esin B. Konyalıhatipoğlu, Dilek Karadoğan, Tahsin Gökhan Telatar, Ünal Şahin

**Affiliations:** 1 Department of Chest Diseases, Recep Tayyip Erdoğan University, School of Medicine, Rize, Türkiye; 2 Department of Public Health, Recep Tayyip Erdoğan University, School of Medicine, Rize, Türkiye

**Keywords:** smoking cessation, motivational interview, hospitalized patients, follow up with phone calls, smoking cessation interventions

## Abstract

**INTRODUCTION:**

‘Teachable moments’, such as inpatient treatment periods, can be turned into opportunities for smokers to acquire healthy living behaviors. This study was conducted to evaluate the outcomes of smoking cessation interventions in an inpatient hospital setting.

**METHODS:**

Data were collected for this single-arm prospective intervention cohort study between October 2021 and March 2022 from hospitalized patients at Recep Tayyip Erdoğan University Training and Research Hospital in Türkiye. Smoker patients received smoking cessation counseling and brief smoking cessation interventions during their hospitalization and were informed about how to apply to our hospital’s smoking cessation outpatient clinic after discharge. They were followed via phone on the 3rd, 5th, and 7th day and the 1st, 3rd, 6th, and 12th month after their discharge, regarding their quit status as well as admissions to smoking cessation clinics. Quitters were confirmed by exhaled air carbon monoxide testing. Logistic regression analysis was performed to evaluate the presence of admission to the emergency department and family physicians at follow-up at 1st year. The model was adjusted in terms of age, sex, presence of malignancy, and education level.

**RESULTS:**

Of the 183 patients included in the study, 163 participants completed periodic follow-up during one year, with quit rate of 47.2%. The rate of anxiety was higher among non-quitters compared to quitters (9.4% vs 1.2%) (p=0.024). Non-quitters were 19 times more likely to have emergency department admissions (AOR=19.64; 95% CI: 8.08–47.68) and eight times more likely to have family doctor visits (AOR=8.43; 95% CI: 4.05–17.53) than quitters.

**CONCLUSIONS:**

This cessation program evaluated the quit rates of hospitalized patients in the first year and revealed that the rate of anxiety was higher in non-quitters compared to quitters. It would be an important approach to include psychiatric support in this practice.

## INTRODUCTION

Tobacco consumption is the leading cause of preventable premature death worldwide. Smoking causes cardiovascular and pulmonary diseases and cancers, and other preventable diseases, disabilities, and deaths^1^. However, smoking cessation assistance has been reported to be neglected even in pulmonary diseases such as asthma and COPD^2^. Hospitals where patients receive inpatient support for treatment are non-smoking areas. Therefore, inpatient treatment is a unique opportunity for patients to stop the smoking addiction^1^. At the same time, it is generally seen that smoking-related disease is a progressive process in inpatients, which strengthens the patient’s desire to quit smoking. In such a motivational environment, healthcare providers need to be trained to provide additional support and behavioral interventions for current smokers^3^. At the same time, the continuation of smoking cessation interventions after discharge is extremely important for the success of the interventions. There are very few international studies on this subject^4^. Research shows that smokers have twice the length of hospitalization as non-smokers^5^. The Ottawa model for smoking cessation (OMSC) is a systematic approach to tobacco-dependence treatment delivered in more than 120 hospital-integrated healthcare settings across Canada. With this model, an 11% improvement was achieved in absolute terms in the smoking cessation rate of patients^5^. Rigotti et al.^6^ who followed patients by telephone on 2, 14, 30, 60, and 90 days after discharge, found that 26% of the patients in the intensive counseling group and 15% of the patients in the standard counseling group were successful in quitting smoking within six months. In a similar study by Regan et al.^7^, the smoking cessation rates of 738 patients hospitalized for various reasons were compared at 2 and 12 weeks after discharge, and the cessation rates were found to be 39% and 29%, respectively.

As mentioned above, smoking cessation interventions for inpatient settings are an unmet need, with only a few examples worldwide. Our aim was to examine the mid-term results of smoking cessation interventions we applied in patients who were active smokers receiving treatment in a hospital setting.

## METHODS

### Sample and setting

The population of our study was hospitalized patients in the inpatient services of Recep Tayyip Erdoğan University Training and Research Hospital between May 2021 and May 2022. For this single-arm prospective follow-up study, we collected data and recruited patients at the hospital between 20 October 2021 and 20 March 2022.

### Sample size calculation

The minimum sample size to evaluate the factors determining the success of smoking cessation was calculated using G*Power 3.1.9.7 software, with an effect size of 30% and a degree of freedom of 1 at 95% power and α=0.05 level, which gave145 participants. The dependent variable was evaluated as ‘quitter’/‘non-quitter’.

### Study design

The design was a single-arm prospective intervention study. First, we identified hospital-wide inpatients who were current smokers, and a total of 251 patients were interviewed.

*Inclusion criteria*

1. Patients hospitalized for at least one day for inpatient care;
2. Patients who were current smokers;
3. Patients aged ≥18 years; and
4. Patients with a cooperation, oriented, and standardized mini mental test score of ≥18.

*Exclusion criteria*

1. Patients receiving inpatient treatment diagnosed with COVID-19;
2. Patients with psychiatric illness; and
3. Patients who did not agree to participate in the study.

Interviews with 183 patients were continued based on the inclusion and exclusion criteria. Participants were randomly selected from inpatient services belonging to surgical or internal medicine branches. Patients identified as smokers during hospitalization were reported to the principal investigator (EBK), and all reported patients were visited and assessed for inclusion in the study. A behavioral smoking cessation model (5As/5Rs) was applied to current smokers who met the inclusion criteria^8^. All patients included in the study were invited to the smoking cessation outpatient clinic after discharge. Periodic phone calls were continued after the discharge of the patients.

After the patients were discharged from the hospital, they were followed up with periodic telephone calls on day 3, 5, and 7, and at month 1, 3, 6 and 12.

### Ethical approval

The research was started after approval from the Recep Tayyip Erdoğan University Training and Research Hospital Ethics Committee.

### Data collection and follow-up

Detailed medical history, medication use, and presence of comorbidities were questioned, and medical records were analyzed through the hospital system. Patients were informed about smoking cessation services, their contact and transportation information was recorded, and they were informed about follow-up after discharge from the hospital. WHO’s short-release interventions (5As/5Rs model) were used in the interviews^9^. Patients who wanted to quit smoking were provided with the 5As support (Ask, Advise, Assess, Assist, and Arrange follow-up). Patients who did not wish to quit smoking were provided with the 5Rs model of assistance which includes counseling and recommendations, and includes Relevance, Risk, Rewards, Roadblocks, and Repetition guidelines^3,10^. Since they were inpatients, it was made clear to the patients that the severity of the disease was usually advanced. Each patient was given a brief medical explanation about their disease. The ‘link’ between their disease and smoking was explained appropriately in the light of scientifically based information.

All patients included in the study were invited to the Smoking Cessation Clinic, and all patients, including those who did not attend the outpatient clinic, were followed up by telephone on day 3, 5, and 7, and at month 1, 3, 6 and 12, after discharge.

### Control remote visits

During the telephone interviews, patients were asked about their smoking cessation status, the daily number of cigarettes, and the presence of a smoker household member. Those who could not quit smoking were asked about the reasons for not quitting. For those who started pharmacological smoking cessation treatments, the use of the treatment and the reasons for not using it, details of any side effects related to the treatment, factors that may prevent smoking cessation during the process, and how they coped with them were recorded in detail. Motivational and behavioral therapy methods were used to ensure that the gains were permanent, with positive reinforcements in case of repeated gains in telephone interviews. In the follow-up interviews conducted for one year, all patients were invited to face-to-face interviews whether or not they were using pharmacological treatment, and the need to continue or change treatment was also evaluated for patients using pharmacological treatment.

Nicotine dependence levels were determined by applying the Fagerström test for nicotine dependence (FTND) to patients who applied to the Smoking Cessation Clinic. FTND has a good level of reliability in determining the level of nicotine dependence with the six items it contains^4^. Clinicians also use FTND to decide on nicotine replacement therapy dosages^11^.

Patients with contraindications to nicotine replacement therapy due to comorbid conditions were consulted accordingly. Patients eligible for pharmacologic treatment were enrolled in the Tobacco Addiction Treatment Tracking System (TUBATIS). Drug assignment was made through this system, and 15 or 25 mg nicotine patch treatment was initiated according to the patient’s identified need^12-14^.

At the end of one year, in addition to the questions asked in other telephone interviews, patients were questioned about their admissions to emergency departments and visits to family physicians, and the number of visits within one year. Patients who declared that they had quit smoking at the last follow-up were invited to the clinic, and exhaled carbon monoxide levels were measured by piCO+^™^ Smokerlyzer^®^ brand carbon monoxide breath test monitor. In the measurements, values ≤5 ppm were considered normal^15^.

### Statistical analysis

Data were analyzed using IBM SPSS Statistics for Windows (Armonk, NY, USA, IBM Corp). Numerical data obtained in the study are presented as mean and standard deviation if normally distributed, and median and interquartile range if not normally distributed.

Categorical data are presented as frequencies and percentages. Relationships between categorical data were evaluated with the chi-squared test and Fisher’s exact test. The distribution characteristics of continuous data were determined by Kolmogorov Smirnov and Shapiro-Wilk tests. The differences between the groups of variables showing normal distribution were evaluated by t-test, and the variables not showing normal distribution were evaluated by Mann Whitney U test. The correlation between the number of visits to the emergency department in the last year and the number of visits to the family physician was determined by Spearman correlation analysis. Logistic regression models were designed to evaluate the factors determining the number of visits to the family physician and admissions to the emergency department in the last year, and were adjusted for age, sex, presence of malignancy, and education level. Results are presented as adjusted odds ratios (AORs) and 95% CI. The significance level was accepted as p<0.05 in all statistical analyses. Successful quitting was defined as sustained abstinence from smoking since the target quit date. Quitting status was determined by self-report and confirmed biochemically at the follow-up at 12 months.

## RESULTS

**Figure 1:**
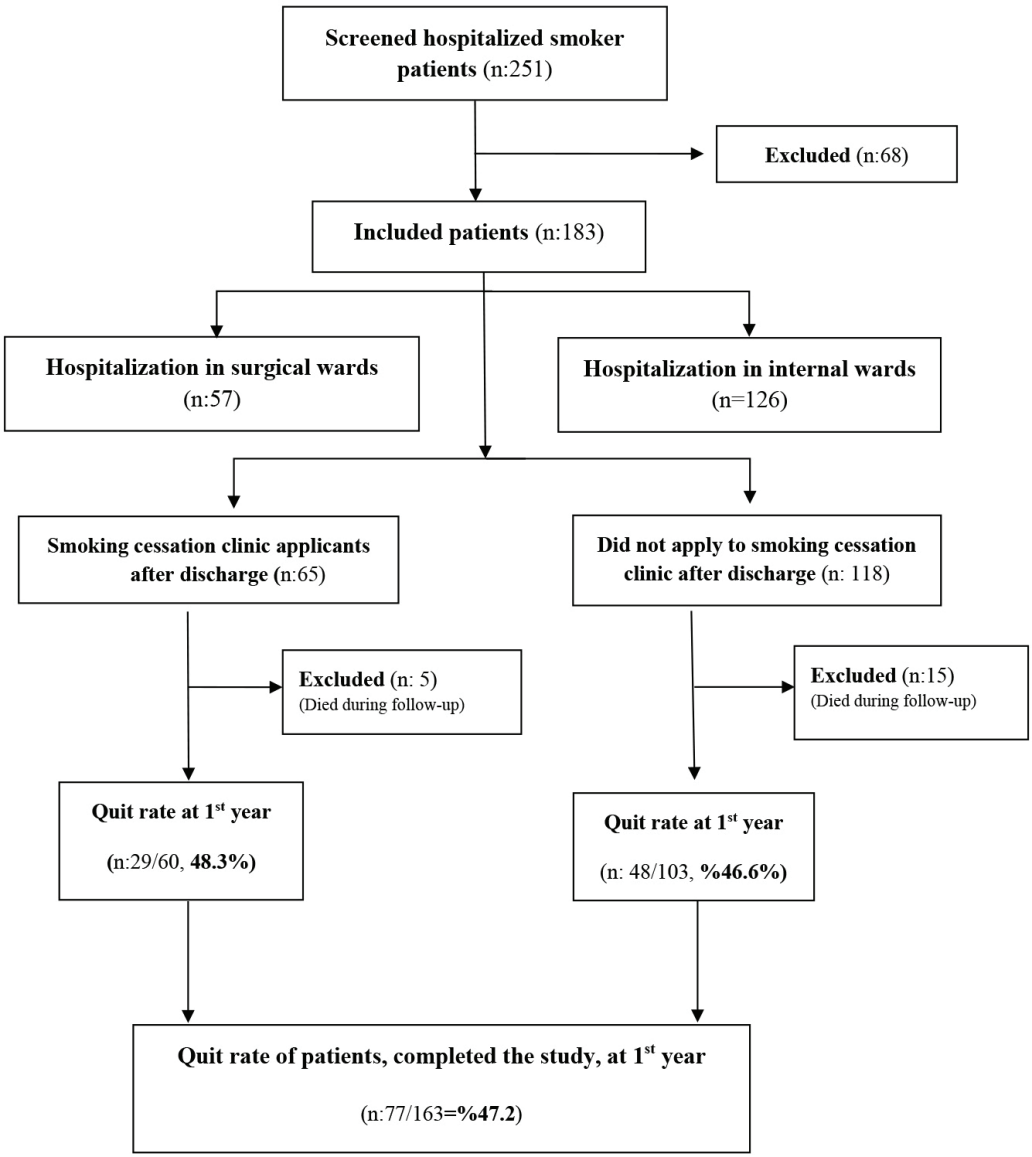
Flowchart showing the distribution of hospitalized smoker patients at Recep Tayyip Erdoğan University Training and Research Hospital included in the study according to smoking cessation outpatient clinic admission and quit rates at 1st year follow-up, between October 2021 and March 2022, Türkiye (N=163)

A total of 183 patients were included in the study, 57 (31.1%) of whom were hospitalized in surgical wards, and 126 (68.9%) were hospitalized in internal medicine departments. Of the 183 patients, 65 (35.5%) applied to our Smoking Cessation Clinic (SCC) after discharge, while 118 (64.5%) did not apply to SCC (Figure 1).

There were 163 participants who completed periodic follow-up during one year; among them, the quit rate was 47.2%. The quit rates were similar between those admitted and not admitted to the SSC, 48.3% (n=29) versus 46.6% (n=48) (p>0.05). The rate of anxiety was higher among non-quitters compared to quitters (9.4% vs 1.2%) (p=0.024) (Table 1). When the total number of visits to the emergency department and family physician at the end of the 1-year follow-up of the participants was analyzed, it was observed that the number of visits to the emergency department (r=0.68) and family physician (r=0.5) of patients who could not quit smoking was considerably higher compared to patients who quit smoking (p<0.001).

**Table 1:** Characteristics of patients who completed the study by quit status at 1st year, Recep Tayyip Erdoğan University Training and Research Hospital, October 2021 and March 2022 (N=163)

*Characteristics*	*Total (N=163) n (%)*	*Patients who quit smoking (N=77) n (%)*	*Patients who did not quit smoking (N=86) n (%)*	*p*
**Age**, mean (SD)	52.97 (14.90)	57.1 (2.07)	56.94 (2.22)	0.200
**Age** (years)				
<45	48 (29.4)	20 (41.7)	28 (58.3)	0.517
45–65	84 (51.5)	40 (47.6)	44 (52.4)	
>65	31 (19.1)	17 (54.8)	14 (45.2)	
**Gender**				
Female	22 (13.4)	13 (59.0)	9 (40.0)	0.377
Male	141 (8.6)	64 (45.4)	77 (54.6)	
**Occupation**				
Self-employment	42 (25.7)	17 (40.5)	25 (59.5)	0.884
Worker	25 (15.3)	10 (40.0)	15 (60.0)	
Official	18 (11.2)	8 (44.4)	10 (55.6)	
Housewife/unemployed/student	17 (10.4)	11 (64.7)	6 (35.3)	
Retired	61 (37.4)	31 (50.8)	30 (49.2)	
**Tobacco pack-years**, median (IQR)	52.9 (45)	50 (45)	50 (30)	0.531
**Tobacco pack-years group**				
<10	16 (9.8)	6 (37.5)	10 (62.5)	0.709
10–20	17 (10.4)	8 (47.1)	9 (52.9)	
>20	130 (79.8)	63 (48.5)	67 (51.5)	
**Education level**				
lİ literate or primary school	75 (46.0)	40 (53.4)	35 (46.6)	0.556
Secondary or high school	69 (42.4)	28 (40.6)	41 (59.4)	
College graduate or higher	19 (11.6)	9 (47.4)	10 (52.6)	
**Fagerström score**, median (IQR)	5.91 (6)	6 (2)	6 (3)	0.248
**Fagerström score**				
Low	17 (10.4)	9 (52.9)	8 (47.1)	0.763
Middle	95 (58.3)	45 (47.3)	50 (52.7)	
High	51 (31.3)	23 (45.1)	28 (54.9)	
**Number of attempts to quit smoking**, median (IQR)	0.4 (0)	1 (0)*	1 (0)**	0.363
**Length of stay** (days), median (IQR)	6.1 (5)	5 (6)	7 (6)	0.440
**Day after discharge applied to SBP** median (IQR)	3.0 (0)	0 (5)	0 (2)	0.168
**Patients who come to the smoking cessation outpatient clinic**	41 (25.1)	24 (58.5)	17 (41.5)	0.103
**Presence of anxiety**	9 (5.5)	1 (1.2)	8 (9.4)	**0.024**

* Range=1–5, mean (SD)=1.29 (0.96).

** Range=1–7, mean (SD)=1.18 (0.94).

Non-quitters were 19 times more likely to have emergency department admissions (AOR=19.64; 95% CI: 8.08–47.68) and eight times more likely to have family doctor visits (AOR=8.43; 95% CI: 4.05–17.53) than quitters (Tables 2 and 3).

**Table 2:** Factors associated with admission to emergency department in the last year, Recep Tayyip Erdoğan University Training and Research Hospital, October 2021 and March 2022 (N=163)

*Factors*	*AOR*	*95% CI*	*p*
Non-quitter (Ref. quitter)	19.60	8.08–47.68	<0.001
Presence of pulmonary disease (Ref. absence)	0.87	0.35–22.18	0.779
Presence of cardiovascular disease (Ref. absence)	1.70	0.56–5.15	0.344
Presence of metabolic diseases (Ref. absence)	0.91	0.32–2.58	0.870
Hospitalization duration (days) (per 1 day increase)	1.02	0.95–1.10	0.441

AOR: adjusted odds ratio; model was adjusted for age, sex, presence of malignancy and education level.

**Table 3:** Factors associated with visits to family physician for various reasons in the last year, Recep Tayyip Erdoğan University Training and Research Hospital, October 2021 and March 2022 (N=163)

*Factors*	*AOR*	*95% CI*	*p*
Non-quitter (Ref. quitter)	8.43	4.05–17.53	<0.001
Presence of pulmonary disease (Ref. absence)	1.39	0.60–3.20	0.439
Presence of cardiovascular disease (Ref. absence)	0.68	0.25–1.87	0.460
Presence of metabolic diseases (Ref. absence)	1.16	0.46–2.94	0.748
Hospitalization duration (days) (per 1 day increase)	1.01	0.94–1.08	0.734

AOR: adjusted odds ratio; model was adjusted for age, sex, malignancy and education level.

## DISCUSSION

Our study examined the effectiveness of smoking cessation interventions for hospitalized patients. Overall, the quit rate in the first year was 47.2%; 48.3% of those who applied to the smoking cessation outpatient clinic, and 46.6% of those who did not apply to the SCC. Moreover, the only significant difference in the quit status of the sample was the presence of anxiety, which was found to be higher in non-quitters compared to quitters. Another important finding was the positive correlation between the number of cigarettes smoked per day and the number of visits to emergency services and family medicine services, as well as the relationship between the number of visits to emergency services and family medicine services and the success of the sample in quitting smoking.

Supporting inpatient interventions for smokers with pharmacologic treatment increases the likelihood of smoking cessation and strengthens the motivation of patients^1^. The effect of depressive mood and anxiety on smoking cessation can be minimized with smoking cessation assistance^16^. Initiating smoking cessation intervention in a hospital setting may help individuals overcome their anxiety^17^. It is important to note that the quit rate was similar in patients who applied to SCC and those who did not. Reinforcing the smoking cessation behavior initiated in the hospital environment with post-discharge follow-up contributes to the behavior change process.

Our study has some similarities and differences with the Ottawa model for smoking cessation (OMSC) regarding sample design and follow-up. In the OMSC model, two groups of OMSC patients were randomized; one group received extra education, counseling, and extra coaching support in the smoking cessation intervention^5,18^. In the model, an absolute increase of 11% (18% to 29%) was found in smoking cessation outcomes with interventions applied to hospitalized patients^5^. The intervention group showed significant reductions in all-cause readmissions, smoking-related re-admissions, and all-cause emergency department visits^5^. Unlike our study, the control arm was composed of non-smokers and hospitalized patients in OMSC. When 2-year mortality, annual smoking-related rehospitalizations, and 2-year all-cause rehospitalizations were taken into consideration, there was no statistically significant difference between the control group and the intervention group. When 6-month smoking cessation data were analyzed, 45 (20.4%) of 221 control patients and 90 (35.2%) of 256 intervention patients quit smoking (p<0.001). In our study, it was observed that those who could not quit smoking were more likely to apply to the emergency department and family physician than those who quit smoking.

As seen in studies, higher smoking cessation success is achieved with smoking cessation interventions performed in hospitalized patients^19^. Continued follow-up of patients after discharge provides high motivational support for the continuation of smoking cessation behavior formed during hospitalization^4,16^.

Unlike the similar studies mentioned above, in our study, we could not initiate an immediate smoking cessation pharmacological treatment option in the intervention arm. We informed them to contact the SCC, and then patients were followed up at more frequent intervals after discharge. The similarity of the cessation rates according to their admission to the SCC indicated that our close follow-up and motivational talks were also very effective. We believe that the most important reason for the high cessation rates we obtained is the frequent follow-up intervals, especially in the early post-discharge period. Initiation and follow-up of such interventions for current smokers to quit smoking in the hospital environment has promising outcomes^1,5^.

The gains from scaling up such interventions are significant globally and individually^1,20^. Our study has shown how feasible it is to implement and integrate such interventions into the health system. Such strategies can significantly increase social welfare by reducing healthcare expenditures and improving individual health.

### Strengths and limitations

The study’s strengths are that it is the first example of smoking cessation interventions applied to hospitalized patients in Türkiye, it has a prospective design, regular and almost complete patient follow-up, complete data, and verification of cessation status with CO measurement. On the other hand, our study has limitations due to its design, such as residual confounding, non-causal design, lack of formal interactions, absence of a non-intervention arm to compare the outcomes, and limited generalizability to other countries.

## CONCLUSIONS

In our previous study, we found that the long-term smoking cessation rate of those who applied to the outpatient smoking cessation outpatient clinic was 20.5%^21^. In the present study, we evaluated the longterm quit rates of hospitalized smokers for whom we implemented smoking cessation interventions, and found a higher quit rate of 47%. Since the hospitalization period is an effective ‘teachable moment’, our study has been a good example in terms of turning it into an advantage for smoking cessation. Our cessation program has demonstrated higher quit rates within the first year among hospitalized smokers compared to quit rates in outpatient settings and also appears to be feasible to incorporate into routine inpatient care.

## Data Availability

The data supporting this research are available from the authors on reasonable request.
